# Risk of cancer in individuals with Lynch-like syndrome and their families: a systematic review

**DOI:** 10.1007/s00432-022-04397-0

**Published:** 2022-10-17

**Authors:** Pandu P. Nugroho, Siti Alyaa S. Ghozali, Daniel D. Buchanan, Mia I. Pisano, Jeanette C. Reece

**Affiliations:** 1 Faculty of Medicine, Universitas Indonesia, Depok, West Java, Indonesia; 2 Melbourne Medical School, The University of Melbourne, Parkville, VIC Australia; 3 Colorectal Oncogenomics Group, Department of Clinical Pathology, Melbourne Medical School, The University of Melbourne, Parkville, VIC Australia; 4 University of Melbourne Centre for Cancer Research, Parkville, VIC Australia; 5 Genomic Medicine and Family Cancer Clinic, Royal Melbourne Hospital, Parkville, VIC Australia; 6 Faculty of Medicine, Dentistry and Health Sciences, The University of Melbourne, Parkville, VIC Australia; 7 Neuroepidemiology Unit, Centre for Epidemiology and Biostatistics, Melbourne School of Population and Global Health, The University of Melbourne, Level 3 207 Bouverie Street, Parkville, VIC 3010 Australia

**Keywords:** Lynch-like syndrome, Lynch syndrome, Colorectal cancer, Extra-colonic cancer, Standard incidence ratio

## Abstract

**Background:**

Lynch-like syndrome (LLS) tumors have similar clinicopathological features to Lynch syndrome (LS) tumors but have no identifiable pathogenic germline mismatch repair gene variant. However, cancer risks in LLS patients and first-degree relatives (FDRs) are not well defined.

**Methods:**

To clarify LLS-associated cancer risks, a systematic review of all studies examining all cancer risks in LLS was performed. Searching of Medline, Embase, Pubmed, Cochrane and CINAHL databases and reference/citation checking identified relevant studies published between January 1, 1980 and February 11, 2021. Joanna Briggs Institute Appraisal Tools assessed the risk of bias.

**Results:**

Six studies (five cohort/one cross-sectional) were eligible for study inclusion. One study found no difference in colorectal cancer (CRC) incidence between LLS and LS patients or CRC risks at aged 70 years. Three studies found CRC incidence in LLS FDRs was higher than the general population but lower than LS FDRs. Two studies showed no difference in CRC diagnosis age between LLS patients and LS patients. Endometrial cancer risks in LLS patients were higher than the general population but lower than LS patients.

**Conclusion:**

Evidence of elevated CRC risks in LLS patients and FDRs supports increased colonoscopy surveillance strategies for LLS patients and FDRs in line with current recommendations for LS. Due to heterogeneity amongst LLS populations, extended intervals between screening may be advised for low-risk families. Studies to resolve the molecular characterization and definition of LLS are needed to clarify cancer risks associated with LLS which in turn may individualize surveillance strategies for LLS patients and families.

**Supplementary Information:**

The online version contains supplementary material available at 10.1007/s00432-022-04397-0.

## Introduction

Lynch syndrome (LS) is an autosomal-dominant inherited syndrome accounting for 2–4% of colorectal and endometrial cancers (Hampel et al. 2006, 2008; Umar et al. 2004). LS is characterized by germline pathogenic variants in one of the DNA mismatch repair (MMR) genes, *MSH2, MLH1, MSH6* and *PMS2* or large deletions in *EPCAM*, causing transition read through hypermethylation of *MSH2* gene promoter (Carethers 2014). Inactivation of MMR genes via a germline pathogenic variant and an acquired somatic mutation (second hit) results in the accumulation of mutations in regions of repetitive DNA during cell replication. This tumorigenesis mechanism leads to tumors with microsatellite instability (MSI), with accompanying loss of MMR protein and high numbers of somatic mutations (hypermutation), collectively referred to as MMR deficiency (Cancer Genome Atlas 2012; Lynch et al. 2015; Rodriguez-Soler et al. 2013).

In addition to LS, there are sporadic causes of tumor MMR deficiency. *MLH1* promoter hypermethylation is the most common cause of MMR deficiency in colorectal cancer (CRC) and endometrial cancer, as characterized by loss of tumor MLH1 and PMS2 protein expression. Distinguishing between *MLH1* methylation and LS-related MMR deficiency is clinically important for secondary cancer risk management and for identifying relatives at risk of cancer.

Lynch-like syndrome (LLS) tumors are considered mimics of LS tumors, also demonstrating MSI, loss of MMR protein expression, and absence of *MLH1* methylation (Carethers 2014; Hampel et al. 2006). However, in LLS, there is an absence of a germline pathogenic variant in one of the *MMR* genes or a somatic *BRAF* V600E mutation (in the absence of *MLH1* methylation) (Carethers 2014; Hampel et al. 2006). LLS tumors constitute up to 70% of patients with MSI and MMR deficiency suspected of having LS.(Carethers & Stoffel 2015; Rodriguez-Soler et al. 2013) The prevalence of CRC cases with LLS in population-based studies was estimated to be 2.5% in Spain (Rodriguez-Soler et al. 2013), and 6% in collective data from the United States, Canada and Australia (Win et al. 2015). However, a Japanese hospital-based study found LLS prevalence in CRC cases to be significantly lower (0.2%) (Chika et al. 2017), which may reflect ethnicity differences between countries.

Several potential mechanisms may underlie LLS, including the presence of an atypical germline pathogenic variant or cryptic mutations in MMR genes not identified by current detection methods or germline pathogenic variants in genes outside MMR genes (Buchanan et al. 2014; Carethers 2014; Pico et al. 2020a). The predominant cause of LLS-related MMR deficiency, responsible for up to 80% of LLS cancers, involves double somatic mutations in the same MMR gene, known as biallelic MMR deficiency (Geurts-Giele et al. 2014; Haraldsdottir et al. 2014; Mensenkamp et al. 2014). Mosaicism of a de novo pathogenic variant may also underlie LLS but is rarely described (Guillerm et al. 2020). Moreover, incorrect immunohistochemistry staining has been identified as contributing factor to LLS diagnoses (Haraldsdottir et al. 2014). Subsequently, LLS cases represent a heterogeneous population comprised of sporadic cases related to biallelic MMR deficiency and inherited cases related to undetected LS or germline pathogenic variants in other DNA repair genes (Clendenning et al. 2011; Haraldsdottir et al. 2014; Liu et al. 2016; Mensenkamp et al. 2014; Morak et al. 2010).

While LS-associated cancer risks are well-known (Dominguez-Valentin et al. 2020; International Mismatch Repair 2021), and there are standard CRC surveillance strategies for LS patients and first-degree relatives (FDRs) (Monahan et al. 2020), LLS-associated cancer risks are unclear, with studies showing conflicting results with regards to the age of CRC diagnosis and risks of CRC and other cancers in LLS patients and FDRs (Bucksch et al. 2020; Overbeek et al. 2007; Pico et al. 2020b; Rodriguez-Soler et al. 2013; Win et al. 2015). Subsequently, no agreed consensus on cancer screening recommendations for LLS patients and FDRs currently exists (Ladabaum 2020). This uncertainty creates challenges for genetic counseling, conferring different degrees of screening for LLS patients and families, ranging from surveillance guidelines for intermediate-risk individuals (Win et al. 2015), to vigorous LS-recommended guidelines (Monahan et al. 2020; Overbeek et al. 2007). Patients with a LLS diagnosis report variability in the interpretation of their diagnosis, cancer risk management advice and how that is communicated to family members (den Elzen et al. 2021). This present study examined the current evidence for cancer risks in LLS patients and FDRs by systematic review of all relevant studies. The findings of this review may inform future surveillance strategies for LLS patients and FDRs.

## Materials and methods

Systematic review of all studies examining LLS-associated cancer risks published from January 1st 1980 to 11th February 2021 was conducted using Preferred Reporting Items for Systematic Reviews and Meta-Analyses (PRISMA) criteria (Moher et al. 2010). The review was registered in PROSPERO (CRD42021238428).

### Search strategy

Medical Subject Headings (MeSH) terms were used to search databases: MEDLINE (Ovid), PubMed, EMBASE, Cochrane Library and Cumulative Index of Nursing and Allied Health Literature (CINAHL). An intersection of MeSH terms related to Lynch-like syndrome (‘Lynch-like’ or ‘suspected Lynch’ or ‘Lynch mimic’ or ‘Lynch like’), Lynch syndrome and hereditary nonpolyposis CRC (HNPCC) were used for the search strategy (Supplementary Table 1). Studies pertaining to cancer risks in LLS patients and families were selected for inclusion by identifying relevant abstracts and screening full-text articles for eligibility by two co-authors (SASG and PPN). Manual reference and citation checking was performed to identify relevant studies not found from the search strategy. Discrepancies between reviewers were resolved by a third reviewer (JCR).

### Eligibility criteria

Inclusion criteria included studies examining cancer risks in CRC patients with confirmed LLS diagnosis following germline MMR gene mutation analysis and MSI analysis or immunohistochemistry (IHC). Papers not published in English, case studies, reviews, editorials, comparative studies and conference abstracts were excluded.

### Data abstraction

Data extraction from full-text articles fulfilling study criteria was performed independently by co-authors (SASG, PPN and MIP) and confirmed by a third reviewer (JCR). A standardized data extraction form was used to summarize participant characteristics and study findings (Alvarez-Lafuente et al. 2004).

### Risk of bias assessment

Two reviewers (SASG and PPN) assessed risk of bias using Joanna Briggs Institute (JBI) Critical Appraisal Checklists according to study type (Soldan et al. 1997) with any disagreements resolved by a third reviewer (JCR). The JBI tool comprises 8–11 checklists (depending on study type), with options of “yes”, “no”, “unclear” or “not applicable” for each question. Studies were classified low risk (> 80%), moderate risk (60–80%), or high risk of bias (< 60%) (Chima et al. 2019; Reece et al. 2021). No studies were excluded based on risk of bias assessment for completeness of reporting all relevant study findings (Shea et al. 2017).

### Narrative synthesis

A comprehensive narrative synthesis of included studies was conducted summarizing main study characteristics and findings (Green et al. 2006). Data findings were analyzed separately before summarizing results for narrative synthesis. A meta-analysis was not possible due to heterogeneity of data across included studies.

## Results

**Fig. 1:**
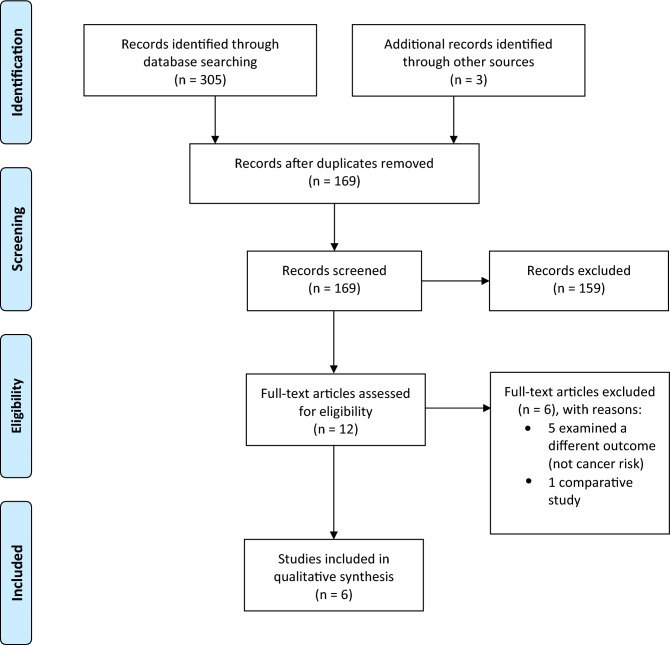
PRISMA flow diagram of inclusion of studies

A total of 1665 studies were identified following searching of five databases (Fig. 1). After removal of duplicates, 169 remaining studies were screened based on title/abstract and 159 studies not fulfilling study criteria were removed. The full-text of 12 remaining studies was assessed for eligibility and five studies were removed for examining a different outcome (not cancer risks) (Giri et al. 2019; Pearlman et al. 2019), different research question (cancer screening adherence and perceived cancer risks in LLS CRC cases (Katz et al. 2016), *UNC5C* mutations in LLS patients (Kury et al. 2014), and validation of an online questionnaire in people undergoing colonoscopy to identify individuals with higher familial and hereditary CRC risks) (Kallenberg et al. 2015). A further study was excluded as it was a comparative study (Mas-Moya et al. 2015). Manually checking reference and citation lists of included papers, identified two further papers for study inclusion, giving a total of six included studies.

The six studies [five cohort (Bucksch et al. 2020; Pico et al. 2020b; Rodriguez-Soler et al. 2013; Win et al. 2015; Xu et al. 2020), and one cross-sectional (Overbeek et al. 2007)] were published between 2007 and 2020 (Table 1). Two studies were from Spain (Pico et al. 2020b; Rodriguez-Soler et al. 2013), one from the Netherlands (Overbeek et al. 2007), Germany (Bucksch et al. 2020), China (Xu et al. 2020), and Australia (using Colon Cancer Family Register data from Australia, US and Canada) (Win et al. 2015).

**Table 1:** Characteristics of included studies

Study	Country	Study type	Cancer type studies	Lynch-like syndrome (LLS) definition	Genetic phenotyping performed	Setting/study period	No. of participants	Characteristics of participants
Overbeek et al. (2007)	The Netherlands	Cross-sectional	Lynch syndrome (LS)-associated cancers, including cancers of the colorectum, endometrium, intestine skin, ovary and urothelial cell carcinomas	Patients with: MSI (microsatellite instability) positive tumor, and No germline mutation in MMR genes, and No MLH1 promoter hypermethylation	Germline mutation analysis was performed on DNA MMR genes obtained from peripheral blood lymphocytes MSI instability was detected using the Bethesda panel of microsatellite markers IHC was performed on tissue sample stained with antibodies against MMR proteins MLH1 promoter methylation was detected using bisulphite treatment on DNA	Data from Department of Human Genetics of the Radboud University Nijmegen Medical Centre, The Netherlands (data from patients that visited Medical Centre between 1997 and November 2005)	18 patients with microsatellite instable (MSI) tumors (LLS) 82 patients with MMR germline mutations (LS)	Mean age at diagnosis was similar in LLS patients and LS patients (44 years) Mean age of patients with *MLH1* promoter hypermethylation was 61 years
Rodriguez-Soler et al. (2013)	Spain	Prospective cohort	Colorectal cancer (CRC), and non-CRC LS-related extra-colonic cancers (pancreas, stomach, ovary, uterus)	Patients with: MSI tumors with identified, and Loss of protein expression of *MSH6*/*MSH6*, isolated loss of *PMS6* protein expression, or Loss of *MLH1* protein expression with no *MLH1* hypermethylation and No germline pathogenic mutation	MSI analysis was performed using BAT26 and NR24 quasi-monomorphic markers IHC analysis of MMR proteins was performed on tumor tissue Germline mutation was assessed using MLPA kit and subsequent DNA sequencing was conducted to identify deletion breakpoints Genetic analysis results were interpreted using ACMG Recommendations for Standards for Interpretation of Sequence Variations (2000) and the InSIGHT database	Data from cohorts in EPICOLON I or II (Spanish nationwide multicenter studies) EPICOLON I; CRC patients newly diagnosed between November 2000 to October 2001. EPICOLON II; CRC patients newly diagnosed between March 2006 and December 2007	Total of 1689 participants 16 LS patients 43 LLS patients, 1630 sporadic CRC patients Families included; First degree relatives (FDRs) of CRC patients with complete pedigree: 13 LS families 25 LLS families 115 families with sporadic CRC	Median age of LLS patients was 66 (55–73) and 69 (51–75) for LS patients. Median age of sporadic CRC patients with 71 (64–78) diagnosis age for FDRs of LS and LLS patients Mean age at diagnosis in LLS FDRs patients was 53.7 ± 16.8 years, compared with FDRs of LS patients was 48.5 ± 14.13; *p* = 0.23 Total—59% male participants; 56% of LLS patients were female; 62.5% of LS patients were female and for sporadic CRC, 40.1% were female
Win et al. (2015)	Australia	Prospective cohort	CRC	CRC proband with tumor with: Loss of expression of MLH1/PMS2 proteins with no *MLH1* hypermethylation or/and *BRAF* V600E mutation Or Loss of expression of MSH2/MSH6 or solitary loss of expression of MSH6 or MSH2, and MSI-H with no identifiable MMR germline mutation	Ten-marker panel and/or IHC was used to assess MMR deficiency by presence of MSI Germline mutation testing were performed on MMR-deficient probands using Sanger sequencing and MLPA Fluorescent alleled-specific PCR assay was used to detect somatic T > A mutation in the *BRAF* V600E gene MethyLight MLH1-M2Methylight reaction was used to identify f MLH1 gene promoter methylation	Data from Colon Cancer Family Registry (Australia, Canada, and the US), between 1997 and 2007	Total of 4853 patients with invasive CRC, with subgroups 4095 probands with MMR-proficient CRC 301 probands with MMR-deficient non-Lynch syndrome 271 probands with suspected Lynch syndrome 186 probands with Lynch syndrome	Mean age of CRC diagnosis in CRC probands; LLS 48.8 ± 12.3 years, and LS 45.0 ± 11.3 years Median age of CRC diagnosis in CRC probands; LLS 47 (IQR 18–74) years, and LS 45.0 (IQR 18–74) years Mean age of CRC diagnosis in FDRs; LLS 57.9 ± 14.8 years, and LS 49.1 ± 13.1 years, *p* < 0.001 Median age (range) of CRC diagnosis in FDRs; LLS 58 (22–90) years, and LS 46 (24–84) years Similar distribution of female and male patients and their FDRs: 51% females had LS 51% females had LLS
Bucksch et al. (2020)	Germany	Prospective cohort	Any cancer and colorectal, urothelial, stomach, small bowel, female breast, ovarian, and endometrial cancer	Tumor with MSI and MMR deficiency (dMMR) but no pathogenic germline mutation in MMR genes (*MLH1, MSH2, MSH6, PMS2*) or *EPCAM*, and No hypermethylation of MLH1 promoter	IHC and/or microsatellite analysis (MSA) was performed on tissue sample to detect dMMR Analysis of MMR germline mutation was performed on dMMR tumors	Data from “German Consortium for Familial Intestinal Cancer” prospective registry. Families with suspicions of LS risks record collected by six institutions Patients were observed during the first colonoscopy or age 25 and observed until diagnosis of cancer of interest, age 80, May 12,2019, or death Median range of 6.8 to 8.1 years, depending on cancer type. Until May 12, 2018 (end of study)	Total 1863 participants; 1200 index patients, 663 at-risk relatives, Subgroups: 594 patients with LLS (320 individuals from families with MMR deficiency in MLH1 protein, 127 in MSH2, 26 in MSH6 and 121 patients were unable to be assigned to a specific MMR protein as only microsatellite analysis but not IHC was performed 116 patients with familial CRC type X (FCCX) 1120 patients with LS	At start of prospective observation, median age (IQR) for any cancer for LLS was 39 (30–46) and for LS was 35 (29–43) for *MLH1*, 36 (30–43) for *MSH2* and 40 (34–48) for *MSH6.* For CRC, median (IQR) was 41 (31–48) for LLS and for LS was 37 (30–45) for *MLH1*, 39 (31–48) for *MSH2* and 42 (36–51) for *MSH6*
Pico et al. (2020, b)	Spain	Prospective cohort	CRC and extracolorectal LS-associated tumor (ovary, endometrium, pancreas, stomach, urinary tract, skin, small intestine, brain, biliary tract)	Patients with tumors with: High MSI (MSI-H), and/or Loss of MMR proteins expression—no *MLH1* promoter hypermethylation No germline mutation in MMR genes or epithelial cell adhesion molecule (EpCAM)	MSI status and/or IHC was performed on tumor tissues of CRC patients Germline mutation analysis was performed on genomic DNA from leucocytes or non-tumor colon tissues DNA sequencing was performed to distinguish deletion breakpoints PCR and direct gene sequencing was used to identify point mutations Genetic analysis results were interpreted using ACMG Recommendations for Standards for Interpretation of Sequence Variations (2000) and the InSIGHT database	EPICOLON III Patients diagnosed from November 2007 and their families followed up until July 2019	Total of 446 patients; 286 LS patients, 160 LLS patients FDRs of CRC patients with complete pedigree included: 1205 FDRs of LS patients for cancer risk analysis 698 FDRs of LLS patients for cancer risk analysis 1126 FDRs of LS patients for prospective study of LS-related cancers 587 FDRs of LLS patients in prospective study of LS-related cancers	LS patients were significantly younger at CRC diagnosis compared to LLS patients Median age (SD) of CRC diagnosis; 48.1 (12.9) years in LS patients, vs 54.9 (14.2) years in LLS patients, *p* = 0.01 Gender: 46.1% of LS patients were female, and 41.3% of LLS patients were female
Xu et al. (2020)	China	Prospective cohort		No pathogenic MMR variant no *BRAF V600* variant OR Carriers of variants of unknown significance	Tumors with *MMR* deficiency variants in *MLH1* or *MLH1* and *PMS2* genes as assessed by IHC were examined for detection of *BRAF* V600 variants (excluded sporadic CRC without a *BRAF* V600 variant) Pathogenicity classification of *MMR* genes was done using the INSIGHT database		81 patients with LLS 47 patients with LS LS families comprised of 142 first- and second-degree relatives LLS families comprised of 210 first- and second-degree relatives	LS patients were significantly younger at CRC diagnosis compared to LLS patients Mean age of diagnosis in LS families was 37.5 ± 8.6 years vs 44.5 ± 13.6 years in LLS families Gender: The mean number of male CRC patients (2.04 ± 1.63) in LLS families was higher than females CRC patients (1.54 ± 1.32)

Five studies had low risk of bias (Bucksch et al. 2020; Overbeek et al. 2007; Rodriguez-Soler et al. 2013; Win et al. 2015; Xu et al. 2020), and one had moderate risk (Table 2; Supplementary Tables 2–3) (Rodriguez-Soler et al. 2013). Strengths and limitations of studies are outlined.

**Table 2:** Critique of included studies

Study	Joanna Briggs Institute risk of bias assessment (study quality)	Strengths/limitations	Recommended surveillance strategy
Overbeek et al. (2007)	100%	Strengths: Rigorous analytic strategies were implemented to characterize patients Limitations: Cross-sectional study design LLS was not defined as extensively as later studies Completeness of patient pedigrees was not a consideration for inclusion	Not discussed
Rodriguez-Soler et al. (2013)	75%	Strengths: SIR were only calculated in families with complete pedigrees Data obtained from general clinics and not from high-risk clinics may be more applicable to general population Limitations: Study had small number of non-colorectal LS-related cancer cases Follow-up time was short	Recommend surveillance should be commenced in patients with LLS and their families at the same age as LS patients recommendations Frequency of CRC screening for should be individualized with longer intervals between screenings for patients with LLS and families
Win et al. (2015)	87.5%	Strengths: Large cohort of FDRs of LS and LLS patients Family history of cancer was systematically collected Application of weights with regards to different sampling strategies across different centers reduced selection bias Limitations: Completeness of patient pedigrees was considered in study inclusion Predominantly Caucasian cases may not reflect other ethnic groups 57% CRC diagnoses in FDRs were based on proband’s or relative’s self-reports	Due to the younger age of CRC diagnosis in LLS probands and the higher risk of CRC in FDRs of LLS probands, results are consistent with age-dependent screening recommendations [that is CRC screening for FDRs of CRC cases to commence screening earlier (40 years of age) compared with 50 years for those without a family history of CRC]
Bucksch et al. (2020)	87.5%	Strengths: Prospective study design mitigated overestimation of cancer risks Limitations: Small sample size Small observation period, especially in patients above 60 years old Possible CRC prevention due to intervention in patients undergoing colonoscopy so might not reflect the natural development of cancer risks Risk of underestimation of SIR in patients with endometrial cancer as population incidences were only available for ICD-10 group C54, not C54.1	Recommended more specific surveillance for LLS patients. Every 3 years, colonoscopy screening for patients with cancer history and their families
Pico et al. (2020; b)	88.9%	Strengths: Large cohort of LS and LLS patients SIR were only calculated in families with complete pedigrees Family pedigrees were carefully updated Prospective study design reduced the risk of overestimating the reported risk of cancers Limitations: Lack of molecular information regarding somatic mutations in LLS patients Possible misreporting or underreporting cancer as not all diagnoses were verified Lack of homogenous follow-up for FDRs for LLS patients may have led to reduction in incident cases in LLS families Prospective follow-up time was short Lack of clear diagnosis in LLS families may have reduced follow-up adherence	Study supported the importance of surveillance for LLS and FDRs, despite lower cancer risks for LS In particular, study advised screening and gynecological examination should be performed in patients and families of patient with LLS until hereditary causes of tumor are ruled out
Xu et al. (2020)	88.9%	Strengths: Cancer diagnoses were confirmed by pathology records Limitations: No MSI analysis was performed (IHC was performed—reported to be 97% concordant with MSI)	Recommended begin colonoscopy screening at an early age (age not specified), plus take family history into account and note high frequency of rectal cancers in LLS. Also recognized cancer screening recommendations are hampered as molecular basis for LLS is not defined

### Lynch-like syndrome definition

There were minor differences in LLS definitions across studies (Table 1). All studies performed MSI analysis except one(Xu et al. 2020), IHC to identify MMR deficiency of MLH1, MSH2, MSH6, and PMS2 and germline analysis to confirm the absence of a DNA MMR gen,(Bucksch et al. 2020; Overbeek et al. 2007; Pico et al. 2020b; Rodriguez-Soler et al. 2013; Win et al. 2015), with three studies confirming the absence of *EPCAM* germline mutations (Bucksch et al. 2020; Pico et al. 2020b; Xu et al. 2020). Four studies included tumors with loss of *MLH1* expression without *MLH1* promoter hypermethylation (Overbeek et al. 2007; Pico et al. 2020b; Rodriguez-Soler et al. 2013; Win et al. 2015). Two studies examined *BRAF* V600E mutations: one study including tumors with or without a mutation (Win et al. 2015), and the other including tumors without a mutation (Xu et al. 2020). No studies examining LLS-associated cancer risks screened for the presence of double somatic MMR mutations (Monahan et al. 2020).

### LLS-related cancer risks

While the present study aimed to examined to examine cancer risks in LLS, this review predominantly focuses on a comparison of cancer risks in LLS compared to LS probands and FDRs, with less focus on cancer risks in LLS compared to the general population. This was a direct result of the study design of most included studies that mainly examined cancer risks in LLS compared with LS. Further, the most common cancer types examined in included studies were LS-related cancers, particularly CRC and endometrial cancer.

### Age of CRC diagnosis

In the Win et al. large population-based study the mean (± SD) age of CRC diagnosis was slightly higher for LLS probands [48.8 (± 12.3) years] than for LS probands [45.0 (± 11.3) years] (Win et al. 2015), consistent with the mean CRC diagnosis age of 44 years for both LLS and LS patients in a smaller Dutch study (Overbeek et al. 2007). Similarly, in a small study (*n* = 34) examining cancer risk perceptions not included in the review, the mean CRC diagnosis age of LLS patients was 47.6 (± 10.9) years but these were a selected subgroup (Katz et al. 2016).

In contrast, a Spanish study by Pico et al. found the median CRC diagnosis age (SD) in LLS patients was significantly higher [54.9 (14.2) years] than LS patients [48.1 (12.9) years, *p* = 0.01] (Pico et al. 2020b). However, this may reflect the small study size compared with the Win et al. (2015) study.

In LLS FDRs, Win et al. found the mean CRC diagnosis age as significantly older (57.9 ± 14.8 years) than LS FDRs (49.1 ± 13.1; *p* < 0.001) (Win et al. 2015), comparable with findings of the smaller Chinese study where the mean age of diagnosis in LLS families was 44.5 ± 13.6 years compared with 37.5 ± 8.6 years in LS families (Xu et al. 2020). However, in the small Spanish study by Rodriguez-Soler et al., the mean CRC diagnosis age between LLS FDRs (53.71 ± 16.8 years) and LS FDRs was not different (48.5 ± 14.13 years; *p* = 0.23) (Rodriguez-Soler et al. 2013).

Notably, confirmation of CRC diagnoses may potentially influence these results. In the Win et al. study, 43% of CRC diagnoses were confirmed by medical records, with proband and family members interviewed to confirm CRC diagnoses, whereas in the two Spanish studies, cancer diagnoses were confirmed by medical records which are likely to be more reliable (Rodriguez-Soler et al. 2013; Xu et al. 2020).

### Risk of any cancer

In the German study by Bucksch et al., the standard incidence ratio (SIR) of any cancer in LLS patients was 2.7 (95% CI 1.2–5.4) which was not significantly different to LS patients (Table 3; SIR = 5.3, 95% CI 3.8–7.3) (Bucksch et al. 2020). However, the cumulative risk of any cancer in LLS patients at 70 years was significantly lower compared to LS patients (log-rank; *p* = 0.043).

**Table 3:** Summary of cancer risks in included studies

Study	No. of participants	Follow-up period	CRC risks in LLS	Extra-colonic cancer risks in LLS
Overbeek et al. (2007)	18 patients with microsatellite instable (MSI) tumors (LLS) 82 patients with MMR germline mutations (LS)	Not applicable (cross-sectional study)	CRC risks were analyzed collectively with LS-related extra-colonic cancers (not separately)	Families of LLS patients had a lower risk of LS-related cancer [colorectal cancer (CRC) and extra-colonic] compared to families of LS patients Families of LS patients fulfilled the Amsterdam II criteria (66%) more than families of LLS patients (11%), the difference was significant (*p* < 0.009)
Rodriguez-Soler et al. (2013)	Total of 1689 participants; 16 LS patients, 43 LLS patients, 1630 sporadic CRC patients Families included; first-degree relatives (FDRs) of CRC patients with complete pedigree: 13 LS families 25 LLS families 115 families with sporadic CRC	Median 8.3 years	The incidence of CRC in FDRs of LLS patients (SIR for LLS = 2.12; 95% CI 1.16–3.56) was significantly lower than the incidence of CRC in FDRs of LS patients (SIR = 6.04; 95% CI 3.58–9.54; *p* < .001)	FDRs of LLS patients have a lower incidence of **non-CRC LS-related cancers** than FDRs of LS patients but the difference is not statistically significant: (SIR = 1.69, 95% CI 0.73–3.34 for FDRs of LLS patients and SIR = 2.81, 95% CI 1.03–6.12; *p* = 0.09 for FDRs of LS)
Win et al. (2015)	Total of 4853 patients with invasive CRC, with subgroups 4095 probands with MMR-proficient CRC 301 probands with MMR-deficient non-Lynch syndrome 271 probands with suspected Lynch syndrome 186 probands with Lynch syndrome	5–15 years follow-up; every 5 years	FDRs of LLS patients have a significantly lower incidence of CRC (SIR = 3.45, 95% CI 2.62–4.57) than FDRs of LS patients (SIR = 9.67, 95% CI 7.10–13.1) The hazard of FDRs of developing CRC was 2.06 (95%CI 1.59–2.67) times higher in LLS CRC patients and 5.37 (95% CI 4.16–6.94) times higher in LS patients compared with FDRs of patients with MMR-proficient CRC	Not examined
Bucksch et al. (2020)	Total 1863 participants; 1200 index patients, 663 at-risk relatives, Subgroups: 594 patients with LLS (320 individuals from families with MMR deficiency in MLH1 protein, 127 in MSH2, 26 in MSH6 and 121 patients were unable to be assigned to a specific MMR protein as only microsatellite analysis but not IHC was performed 116 patients with familial CRC type X (FCCX) 1120 patients with LS	Median follow-up time varied depending on cancer type and patient subgroup: 5.9–6.9 person-years in LLS patients 6.0–8.1 person-years in LS patients	CRC incidence in LLS patients (SIR = 14.8; 95%CI 5.4–32.2) was not significantly different to LS patients (SIR = 24.3, 95% CI 16.2–35.1) LLS patients had a lower CRC risk at aged 70 years (21.0%, 95% CI 9.9–41.3%) compared to LS patients (40.9%, 95% CI 28.3–56.4%), but the difference was not significant: log-rank, *p* = 0.102) CRC incidence in males with LLS (SIR = 46.1; 95% CI 12.6–118.1) and LS (SIR = 25.2, 95% CI 13.4–43.2) was higher than the general population and not significantly between LLS and LS CRC incidence in the females with LLS was not higher than the general population (SIR = 6.3; 95% CI 0.8–22.7) CRC risks in index LLS patients were higher than CRC risks in at-risk LLS relatives at 70 years: CRC risk in index LLS patients at aged 70 was 50% (95%CI 19.6%-88.9%) compared to 13.2% (95% CI 4.4–35.9%) in at-risk LLS relatives (*p* = 0.027)	*Incidence in LLS patients was not significantly different to LS patients for:* **Any cancer** (LLS patients: SIR = 2.7; 95% CI 1.2–5.4 and for LS patients: SIR = 5.3, 95%CI 3.8–7.3), **Stomach** cancer (LLS patients: SIR = 6.1; 95%CI 1.7–15.7 and for LS patients: SIR = 6.1, 95% CI 2.8–11.6) ** Urothelial** cancer (LLS patients: SIR = 6.6; 95% CI 1.8–16.8 and for LS patients: SIR = 20.6, 95% CI 13.7–29.8) *Incidence in LLS patients was not higher than the general population for:* **Small bowel** cancer (LLS patient: SIR = 11.9, 95% CI 0.3–66.3 and LS patients: SIR = 126.0, 95% CI 79.9–189.0) *Incidence in ****females**** with LLS was higher than the general population for:* **Endometrial** cancer SIR (14.5; 95%CI 4.7–33.8), but was lower than for LS patients (SIR = 57.8, 95% CI 36.7–86.8) **Urothelial** cancer (SIR = 18.2; 95% CI 3.8–53.2), and with no difference in incidence with LS patients (SIR = 27.7, 95% CI 12.7–52.6) *Incidence in ****males**** with LLS was higher than the general population for:* Stomach cancer (SIR = 7.7; 95% CI 1.6–22.5), and with no difference in incidence with LS patients (SIR = 5.2, 95% CI 1.7–12.2) Cumulative cancer risk at 70 years was higher in LS patients compared to LLS patients for any cancer (log-rank; *p* = 0.043, **urothelial** (log-rank; *p* = 0.015), **small bowel** (log-rank; 0.004), and **endometrial** cancer (log-rank; *p* = 0.002) **Endometrial** cancer risks in index LLS patients were lower (4.0%; 95% CI 1.0–15.3%) than cancer risks in at-risk LLS relatives (23.1%; 95% CI 8.1–55.8%) at 70 years (*p* = 0.01) **Urothelial cancer** risk in women from LLS families with MSH2 protein deficiency was higher than with MLH1 or MSH6 protein deficiency (*p* = 0.003)
Pico et al. (2020; b)	Total of 446 patients 286 LS patients 160 LLS patients FDRs of CRC patients with complete pedigree included: 1205 FDRs of LS patients for cancer risk analysis 698 FDRs of LLS patients for cancer risk analysis 1126 FDRs of LS patients for prospective study of LS-related cancers 587 FDRs of LLS patients in prospective study of LS-related cancers	Median 3 years (IQR 1–6 years)	Incidence of CRC was significantly lower in FDRs of LLS patients compared to FDRs of LS patients but higher than the general population (Spanish national registries) SIR for CRC in FDRs of LLS patients was 2.08; 95% CI 1.56–2.71; SIR for CRC in FDRs of LS patients was 4.25; 95% CI 3.67–4.90 vs.; *p* < 0.001) In a prospective follow-up, the appearance of new cases of CRC was lower in LLS patients and their families (0.5%) compared with LS patients and their families (1.9%); *p* = 0.019	Incidence of **non-colorectal LS-related** cancer was significantly lower in FDRs of LLS patients (SIR = 2.04, 95% CI 1.44–2.80; *p* < 0.001) compared to FDRs of LS patients (SIR = 5.01, 95% CI 4.3–5.8) but higher than the general population (where complete information on age and tumor history was available) In non-colorectal LS-related cancers: Frequency of **endometrial** cancer was significantly lower in FDRs of LLS patients (20.0%) compared to FDRs of LS patients (48.4%; *p* = 0.001) Frequency of **pancreatic** cancer was significantly higher in FDRs of LLS patients compared to FDRs of LS patients (LS = 3.3% vs. LLS = 15.0%; *p* = 0.003) There were no significant differences in the frequencies of **ovarian, stomach, urinary tract, skin, small intestine, brain and biliary tract** cancer between LLS families and LS families In prospective follow-up, LLS families had a lower incidence of new LS-related cancer cases compared to LS families (log-rank; *p* = 0.0001) In prospective follow-up, the appearance of new cases of non-CRC LS-associated tumors was lower in LLS patients and their families (0.3%) compared with LS patients and their families (2%); *p* = 0.006
Xu et al. (2020)	81 patients with LLS 47 patients with LS LS families comprised of 142 first- and second-degree relatives LLS families comprised of 210 first- and second-degree relatives	Follow-up: LS group = 28.8 ± 29.1 months; LLS group = 38.6 ± 24.9 months (2–3 monthly)	In prospective follow-up, there was no difference in no. of metachronous CRCs between LS patients [34.0% (16/47)] and LLS patients [38.3% (31/81)] Left-sided CRCs were significantly lower in LLS families [70.4% (57/81)] than LS families [91.5% (43/47)] The number of rectal tumors was higher in LLS families [25.9% (21/81)] than in LS families [10.6% (5/47)]	The number of extra-colonic cancers in LLS families was significantly higher (1.6 ± 1.4) than in LS families (1.1 ± 1.4) In LLS families, 29 probands developed 29 cases of **primary extra-CRCs: 8 gastric, 6 endometrial, 4 small intestinal, 4 breast, 2 prostate, 2 ovarian and 1 case each of ureteral carcinoma, renal cancer, and pancreatic cancer** In LS families, 11 probands developed 15 **primary extra-CRCs: 5 endometrial and gastric cancer cases, 2 small intestinal cancer and 1 case each of ovarian, breast and cutaneous cancer**

### CRC risks

Bucksch et al. found no difference in CRC incidence in LLS patients (SIR = 14.8; 95% CI 5.4–32.2) compared with LS patients (SIR = 24.3, 95% CI 16.2–35.1) or CRC risks in LLS patients at age 70 (21.0%, 95% CI 9.9–41.3%) compared to LS patients (40.9%, 95% CI 28.3–56.4%; log-rank, *p* = 0.102) (Bucksch et al. 2020). However, this study may be underpowered to identify differences given the small study size, as evidenced by the wide confidence intervals and the inability to find a difference in lifetime CRC risk between LS and Familial Colorectal Cancer Type X (FCCTX) which is inconsistent with a previous study (Samadder et al. 2017).

In males with LLS, Bucksch et al. found CRC incidence was higher (SIR = 25.2, 95% CI 13.4–43.2) than females (SIR = 6.3; 95% CI 0.8–22.7), consistent with Xu et al. where the mean number of males with CRC in LLS families was higher (2.04 ± 1.63) than females (1.54 ± 1.32) (Xu et al. 2020). Similarly, higher incidences of CRC cases in males have been reported in population-based cancer registry data from Europe, Australia and the US (Bray et al. 2018), and in LS families (Sehgal et al. 2014).

Prospective follow-up of participants after their first colonoscopy or at 25 years by Bucksch et al. found LLS index patients had higher CRC risks at 70 years (50%, 95% CI 19.6–88.9%) than their at-risk relatives (13.2%, 95% CI 4.4–35.9%; *p* = 0.027).(Bucksch et al. 2020) Further, in the Pico et al. prospective follow-up study, CRC incidence was lower in LLS patients and families (0.5%) compared with LS patients and families (1.9%; *p* = 0.019) and LLS patients were at significantly lower risk of CRC than LS patients (log-rank; *p* = 0.0001) (Pico et al. 2020b).

In an earlier study, Overbeek et al. found LLS families carried a lower CRC risk (11%) compared with LS families (66%; *p* < 0.009) (Overbeek et al. 2007). Three later studies found CRC incidence in LLS FDRs was higher than the general population but lower than LS FDRs; in Rodriguez-Soler et al. and Pico et al. studies the CRC SIR was 2.1 in LLS FDRs (Pico et al. 2020b; Rodriguez-Soler et al. 2013), and 6.04 (95% CI 3.58–9.54) (Rodriguez-Soler et al. 2013), and 4.25 (95% CI 3.67–4.90; *p* < 0.001) (Pico et al. 2020b), respectively, in LS FDRs. Consistent with these findings, the larger Win et al. study found CRC incidence was lower in LLS FDRs (SIR = 3.45 95% CI 2.62–4.57) than LS FDRs but SIRs were higher in magnitude (SIR = 9.67, 95% CI 7.10–13.1) (Win et al. 2015). Notably, both Spanish studies comprised small family cohorts so there was potentially selection bias. Pico et al. also included LS FDRs regardless of whether or not they carried MMR mutations (Pico et al. 2020b), where Rodriguez-Soler et al. only performed genetic testing in people with cancers which may influence study findings (Rodriguez-Soler et al. 2013). In the Win et al. study, as 57% of CRC diagnoses in FDRs were self-reported (Win et al. 2015), if a proportion of these are false-positive diagnoses, this may underestimate true associations.

Stratification by tumor location in the small Chinese cohort found significantly less left-sided CRCs and significantly more rectal tumors in LLS families compared with LS families (Xu et al. 2020). Notably, the study by Mas-Moya et al. also found LLS patients were more likely to have right-sided colon cancers and not have synchronous and metachronous tumors (Mas-Moya et al. 2015), but as this was a comparative study it was not included in the systematic review.

### Risks of extra-colonic Lynch syndrome-related cancers

Two small Spanish studies showed conflicting results in extra-colonic LS-related cancer (ECLSRC) risks. Pico et al. found the incidence of ECLSRCs in LLS FDRs was higher than the general population (SIR = 2.04, 95% CI 1.44–2.80) but lower than LS FDRs (SIR = 5.01, 95% CI 4.26–5.84; *p* < 0.001) (Pico et al. 2020b). However, no difference between LLS families and LS families in frequencies of ovarian, stomach, urinary tract, skin, small intestine, brain and biliary tract cancer were found (*p* < 0.05). Similarly, Rodriguez-Soler et al. found the incidence of ECLSRCs in LLS FDRs was not higher than the general population (SIR = 1.69, 95% CI 0.73–3.34) and lower than LS FDRs (SIR = 2.81, 95% CI 1.03–6.12) (Rodriguez-Soler et al. 2013).

In a prospective follow-up, Pico et al. found the appearance of new cases of ECLSRCs after an index case diagnosis was lower in LLS patients and families (0.3%) compared with LS patients and families (2%; *p* 0.006) (Pico et al. 2020b).

### Endometrial cancer risks

Bucksch et al. found endometrial cancer incidence in LLS patients (SIR = 14.5; 95% CI 4.7–33.8) was significantly lower than in LS patients (SIR = 57.8, 95% CI 36.7–86.8) (Bucksch et al. 2020), consistent with increased LS-related endometrial cancer risks (Hampel et al. 2006). Cumulative endometrial cancer risk at 70 years was also significantly lower in LLS patients compared to LS patients (log-rank; 0.002).

While Pico et al. found the frequency of endometrial cancer in LLS FDRs was high (20%), it was significantly lower than LS FDRs (48.4%; *p* = 0.001) (Pico et al. 2020b). Bucksch et al. also found endometrial cancer risks in index LLS patients at 70 years were lower (4.0%, 95% CI 1.0–15.3%) than LLS relatives (23.1%, 95% CI 8.1–55.8%; *p* = 0.01) (Bucksch et al. 2020).

### Urothelial cancer risks

Bucksch et al. found the incidence of urothelial cancer in LLS patients was higher than the general population (SIR = 6.6; 95% CI 1.8–16.8) and not significantly different to LS patients (SIR = 27.7, 95% CI 12.7–52.6); however, cumulative risk at 70 years was lower in LLS patients compared to LS patients (log-rank; *p* = 0.015) (Bucksch et al. 2020). Further, women from LLS families with MSH2 protein deficiency had higher urothelial cancer risks than from LLS families with MLH1 or MSH6 protein deficiency (*p* = 0.003) (Bucksch et al. 2020).

### Pancreatic cancer risks

Pico et al. found 6 of the 40 extra-colonic tumors in LLS FDRs were pancreatic tumors (15%), significantly higher than in LS FDRs (3.3%; *p* = 0.003) (Pico et al. 2020b). Similarly, Xu et al. found 1 pancreatic cancer case in 29 extra-colonic tumors (3.5%) in LLS families and no cases in LS families (Xu et al. 2020).

### Stomach cancer risks

One study reporting stomach cancer incidence in LLS patients found the incidence (SIR = 6.1; 95% CI 1.7–15.7) was similar to LS patients (SIR = 6.1, 95% CI 2.8–11.6) (Bucksch et al. 2020). Stomach cancer incidence was also higher in males with LLS (SIR = 7.7; 95% CI 1.6–22.5), similar to males with LS (SIR = 5.2, 95% CI 1.7–12.2).

### Small bowel cancer risks

Bucksch et al. found the incidence of small bowel cancer in LLS patients was comparable to the general population (SIR = 11.9, 95% CI 0.3–66.3) and significantly lower than in LS patients (SIR = 126.0, 95% CI 79.9–189.0) (Bucksch et al. 2020). However, the number of cases was small as indicated by the wide confidence intervals. Nevertheless, cumulative cancer risks at 70 years were significantly lower in LLS patients than LS patients (log-rank; 0.004).

### Surveillance recommendations

Rodriguez-Soler et al. recommended cancer surveillance for LLS families to be commenced at similar age as LS families albeit with longer intervals between surveillance, given the age of CRC diagnosis between LS and LLS patients was similar while the CRC risks were lower in LLS families than LS families (Table 3) (Rodriguez-Soler et al. 2013). Pico et al. recommended cancer screening for FDRs of LLS patients and gynaecologic surveillance of female LLS patients and FDRs, due to higher CRC and gynaecological cancer risks in LLS compared with the general population (Pico et al. 2020b). Win et al. suggested compliance with general age-dependent screening recommendations but for CRC screening of FDRs of CRC cases to commence screening earlier (40 years) due to the younger age of CRC diagnosis in LLS probands and higher CRC risk in LLS FDRs (Win et al. 2015). Xu et al. recommended commencing screening at an early (unspecified) age, taking family history and higher rectal cancer risks into consideration (Xu et al. 2020).

## Discussion

Clinicopathological characteristics of LLS tumors are similar to LS tumors. However, while cancer risks in LS patients and FDRs are well established, LLS-related cancer risks are less certain (Buchanan et al. 2014; Pico et al. 2020a). The present study represents the first systematic review to examine cancer risks in individuals with LLS and families to help address clinically related ambiguities and surveillance strategies. Review of six included studies (five cohort and one cross-sectional) found CRC incidence in LLS patients was similar to LS patients (Bucksch et al. 2020); however, CRC incidence in LLS FDRs was lower than LS FDRs but higher than the general population (Bucksch et al. 2020; Pico et al. 2020b; Rodriguez-Soler et al. 2013; Win et al. 2015). Studies also found the age of CRC diagnosis was comparable between LS and LLS patients (Overbeek et al. 2007; Win et al. 2015), and FDRs (Rodriguez-Soler et al. 2013), but these findings were inconsistent across studies (Pico et al. 2020b; Rodriguez-Soler et al. 2013; Xu et al. 2020).

Notably, two studies found endometrial cancer risks in LLS patients and FDRs were lower than LS patients and FDRs but significantly higher than the general population (Bucksch et al. 2020; Pico et al. 2020b). The incidence of urothelial and stomach cancer in LLS patients was also high, comparable to LS patients, advising vigilance for related symptoms, particularly in females with MSH2 protein deficiency due to associated risks (Bucksch et al. 2020). While two studies indicated pancreatic cancer risks may be elevated in LLS families (Pico et al. 2020b; Xu et al. 2020), surveillance for pancreatic cancer is currently only recommended for high-risk individuals in a research setting (Pico et al. 2020b).

Critically, it is important to consider findings of this review in the context of the shortcomings of the current definition of the molecular phenotype of LLS. Although there are a number of potential causes of LLS, there is the possibility of heterogeneity across different LLS populations due to the differing definitions used in studies examining LLS-related cancer risks (Clendenning et al. 2011; Haraldsdottir et al. 2014; Liu et al. 2016; Mensenkamp et al. 2014; Morak et al. 2010). In particular, studies suggest up to 80% of cases suspected to have LLSs have double somatic MMR mutations (biallelic MMR deficiency) (Geurts-Giele et al. 2014; Haraldsdottir et al. 2014; Mensenkamp et al. 2014), with the most recent NCCN guidelines (Genetic/Familial High-Risk Assessment: Colorectal, version 1.2021) not available prior to the included studies, recommending screening for double somatic mutations in people with unexplained MMR deficiency. Importantly, Pearlman et al. also found people with LS were more likely to be afflicted with LS-related tumors than people with double somatic mutations and also meet Amsterdam II criteria (Pearlman et al. 2019). Other studies indicated current analytic techniques may not identify cryptic or complex *MMR* genetic variants (Clendenning et al. 2011; Morak et al. 2010; Pope et al. 2021), or variants in MMR regulatory regions rarely screened (Liu et al. 2016). However, as studies examining LLS-related cancer risks did not screen for the presence of double somatic mutations or these variants, we could only report the findings from available studies and acknowledge that LLS populations comprise a heterogeneous population with a large number of cases with double somatic mutations. Future studies should aim to examine the LLS-related cancer risks for patients with confirmed double somatic MMR mutations.

Clarifying the definition of LLS may be possible through advances in genetic testing strategies, as indicated by a recent study using a gene panel designed ad hoc in combination with pathogenicity variant assessment to identify potentially causal LLS genes (deleterious MMR mutations) (Damaso et al. 2020). This is particularly pertinent for older studies included in the review using less accurate genetic technology than newer studies (Overbeek et al. 2007; Rodriguez-Soler et al. 2013), but as three of the six included studies were published within the last year (Bucksch et al. 2020; Pico et al. 2020b; Xu et al. 2020), the findings of this review are likely to be relevant for some time until there is a broader implementation of tumor testing to resolve LLS diagnosis.

Despite the acknowledged limitations regarding included studies failing to identify double somatic mutations in LLS patients, our findings support regular colonoscopy screening of index cases in LLS patients and implementing early cancer screening in LLS FDRs due to earlier ages of CRC diagnoses and elevated CRC risks in LLS patients and FDRs (Bucksch et al. 2020; Pico et al. 2020b; Rodriguez-Soler et al. 2013; Win et al. 2015). Monahan et al. currently recommends 2-yearly colonoscopy screening for CRC (as opposed to FIT/FOBT) for LLS individuals with unexplained MMR deficiency that do not have double somatic mutations and FDRs up to 75 years commencing at 25 years, consistent with recommendations for *MLH1* and *MSH2* pathogenic variant carriers (Monahan et al. 2020). However, as two studies found the age of CRC diagnosis was older in LLS patients (Pico et al. 2020b), and LLS FDRs (Win et al. 2015), than LS patients and FDRs, colonoscopy surveillance could potentially commence later, consistent with Monahan et al. recommendations for *MSH6* and *PMS2* pathogenic variant carriers (at 35 years) (Monahan et al. 2020). Further, longer intervals between colonoscopy may be advised due to lower CRC risks in LLS FDRs than LS FDRs (Bucksch et al. 2020; Pico et al. 2020b; Rodriguez-Soler et al. 2013; Win et al. 2015), consistent with the Buchanan et al. review that favored increased intervals > 2 years between screening (Buchanan et al. 2014). Importantly, family history of cancer needs to be considered and clinical recommendations devised based on individual LLS families which may result in personalized colonoscopy surveillance strategies for certain LLS families (Bucksch et al. 2020; Xu et al. 2020). Moreover, Monaghan et al. recommend individuals with double somatic MMR mutations be followed up based on family history of cancer, not LLS guidelines (Monahan et al. 2020).

Strengths of this study include the strict adherence to PRISMA guidelines (Page et al. 2021), and strict study eligibility criteria to identify and include relevant articles. Search strategies, data extractions and risk of bias assessment were performed by two independent reviewers, with a third person resolving any discrepancies. Included studies used appropriate and uniform methods for identifying MMR-deficient tumors and genetic phenotyping to define LLS and LS patients (Bucksch et al. 2020; Overbeek et al. 2007; Pico et al. 2020b; Rodriguez-Soler et al. 2013; Win et al. 2015). Consequently, comparisons across studies are unlikely to be affected by the methodology used across studies. Further, five included studies had a low risk of bias (Bucksch et al. 2020; Overbeek et al. 2007; Pico et al. 2020b; Win et al. 2015; Xu et al. 2020).

The study is limited by the scarcity of relevant studies examining LLS-related cancer risks eligible for inclusion and heterogeneity between included studies related to cancer diagnosis assessments, follow-up times and subgroups of participants. As mentioned, none of the included studies screened for the presence of double somatic mutations and, especially for older studies, the extent of germline MMR gene testing to identify more challenging mutations such as the inversion of exons 1–7 in *MSH2* is unclear. Consequently, it was not feasible to conduct a meta-analysis to quantitate cancer risks. While two studies examined prospective follow-up (Bucksch et al. 2020; Pico et al. 2020b), only Bucksch et al. reported intensified colonoscopy surveillance of participants (Bucksch et al. 2020), and Pico et al. acknowledged decreased adherence to follow-up in LLS FDRs, potentially due to an unclear LLS diagnosis (Carayol et al. 2002), may limit study findings (Pico et al. 2020b). Median follow-up was relatively short (3–8.3 years) (Bucksch et al. 2020; Pico et al. 2020b; Rodriguez-Soler et al. 2013; Win et al. 2015), so cancers may not have been detected during this time. Further, some studies had only small numbers of cases, particularly for extra-colonic cancers (Bucksch et al. 2020; Pico et al. 2020b; Rodriguez-Soler et al. 2013), influencing reported cancer risks and screening recommendations. Study findings may also not be generalizable to non-Caucasian populations as the review did not include any studies from non-Westernized countries.

## Conclusion

Systematic review of relevant articles in the literature found LLS patients and FDRs are at increased risk of developing CRC and potentially at an earlier age to the general population, supporting recommendations of increased colonoscopy surveillance for LLS patients and FDRs, in line with LS guidelines. However, commencing screening at a later age and extending the time between screening may be considered for low-risk LLS families. Future studies focused on molecularly characterized LLS cases will help resolve the germline and somatic etiological heterogeneity and enable studies to refine cancer risks for double somatic MMR mutation cases, leading to more personalized surveillance strategies for individual patients and families.

## Supplementary Information

Below is the link to the electronic supplementary material.

Supplementary file1 (DOC 30 KB)

## Data Availability

All data are available for this study.
