# Supplementary material for: Risk of cancer in individuals with Lynch-like syndrome and their families: a systematic review
Source: J Cancer Res Clin Oncol. 2022 Oct 17;149(1):25–46. doi: 10.1007/s00432-022-04397-0 (PMC9889410; doi:10.1007/s00432-022-04397-0)
Supplement: Supplementary file 1 — Supplementary file1 (DOC 30 KB) [file 432_2022_4397_MOESM1_ESM.doc]

PRISMA flow diagram of inclusion of studies

**Screening**

**Included**

**Eligibility**

**Identification**

Records identified through database searching
(n = 305)

Additional records identified through other sources
(n = 3)

Records after duplicates removed
(n = 169)

Records screened
(n = 169)

Records excluded
(n = 159)

Full-text articles assessed for eligibility
(n = 12)

Full-text articles excluded (n = 6), with reasons:

- 5 examined a different outcome (not cancer risk)
- 1 comparative study

Studies included in qualitative synthesis
(n = 6)
